# Editorial: Hormones and biostimulants in plants: physiological and molecular insights on plant stress responses

**DOI:** 10.3389/fpls.2024.1413659

**Published:** 2024-05-15

**Authors:** Mohammad Shah Jahan, Md Mahadi Hasan, Md Atikur Rahman

**Affiliations:** ^1^ Department of Horticulture, Sher-e-Bangla Agricultural University, Dhaka, Bangladesh; ^2^ State Key Laboratory of Herbage Improvement and Grassland Agro-Ecosystems, College of Ecology, Lanzhou University, Lanzhou, Gansu, China; ^3^ Grassland and Forage Division, National Institute of Animal Science, Rural Development Administration, Cheonan, Republic of Korea

**Keywords:** plant growth regulators, cross talk, phytohormones, ABA, ethylene, stress tolerance

Biotic and abiotic stress signals are critical limitation of growth, productivity, and quality of cultivated crops. These challenging environmental conditions caused by biotic and abiotic stress factors disrupt the normal physiological, biochemical, and molecular activities of plants and weaken their natural defense mechanisms, rendering them more vulnerable to stress. The biostimulants are vital keywords that have been used in this Research Topic. A plant biostimulant is a type of substance or microorganism applied to plants to enhance nutritional benefits, quality, plant fitness, and abiotic stress tolerance (Du Jardin, 2015). The term ‘biostimulant’ was first defined by Kauffman et al. (2007) in a peer-reviewed article, where it was stated that ‘biostimulants are materials that promote plant growth when applied in low quantities.’ Thus, the applied dose of a biostimulant is one of the vital factors for its effectiveness. Biostimulants are composed of a variety of formulations and different ingredients. Kauffman et al. (2007) categorized biostimulants into three major groups based on their source and compositions, including amino acid-containing products (AACP), humic substances (HS), and hormone-containing products (HCP). Seaweed extracts and active plant growth substances such as auxins, cytokinins, or their derivatives belong to the category of HCPs (Du Jardin, 2015). The effective biostimulants including phytohormones has recently been widely applied to enhance crop quality and mitigate the adverse impacts of environmental stresses (Gu et al., 2022; Altaf et al., 2023). Furthermore, the interaction between these hormones and biostimulants with stress conditions leads to modifications of gene’s activity, metabolic pathways, and antioxidant systems within plants. Therefore, exploring the underlying physiological and molecular mechanisms, interplay between hormones and biostimulants in plants under stress response are crucial for plant improvement. These combined studies uncover significant achievements of plant stress tolerance and sustainable agricultural production.

This current Research Topic includes five regular research articles and three review articles, focusing particularly on phytohormones or biostimulants, as well as abiotic (drought, salinity, heat) and biotic stress responses in plants. The majority of the articles published on this topic are related to phytohormones more than to biostimulants. Three papers emphasize plant defense responses influenced by interactions with phytohormones and biostimulant strategies to improve plant stress tolerance. For example, melatonin (N-acetyl-5-methoxytryptamine) is considered a biostimulant rather than a phytohormone. It is a versatile molecule that serves a variety of roles in plants. Recently, the melatonin has been uncovered as a key natural safeguard for crop plants, effectively alleviating both abiotic and biotic stressors in diverse plant species (Jahan et al., 2023). Furthermore, the coordination of MT and ABA is crucial in various physiological processes including stomatal characteristics, wax deposition, delayed leaf ageing, and growth in plants has been documented. Ali et al. explored a link between MT and ABA in Solanaceous vegetable crops is critical for enhancing farming practices and developing crop quality. Furthermore, they hypothesized that it might be beneficial to incorporate an understanding of the MT-ABA coordination in different breeding programs. The exogenous supplementation of MT and ABA could be effective strategies for improving horticultural crop and further these can be useful to the plant breeders (Arnao and Hernández-Ruiz, 2019). Furthermore, agonists that target only the ABA receptor hold significant promise in agriculture, even though thorough physiological studies in crops are lacking. Jiménez-Arias et al. investigated how AMF4 protects tomato plants during drought stress by enhancing CO_2_ assimilation, increasing proline accumulation, and improving the content of specific macronutrients. They also stated that the molecular mechanism of AMF4 activity involves in regulating ABA-responsive genes and the promotion of stomatal closure to reduce osmotic stress in tomato plants.

Brassinosteroids (BRs) are a group of polyhydroxylated steroidal important PHs that modulates a variety of physiological processes, including plant growth, pollen tube development, leaf winding and subscale, root repression, ripening of fruit, proton pumping event, xylem differentiation, chlorophyll level, and gene expression in diverse plant species (Ahammed et al., 2013). Moreover, recent findings have demonstrated that they possess the ability to protect plants against various biotic and abiotic stressors, such as diseases, harmful compounds, water scarcity, high temperatures, and salinity. Zhang et al. focused on the involvement of BRs in resiliencing several abiotic stresses that lead yield and quality in horticultural crops. These strategies open new dimension of research to ensure yield and quality of diverse horticultural plants. Furthermore, the advancement of understanding and the potential application of BRs in horticultural crops suggest promising approaches for developing qualitative and quantitative traits in edible crops.

Whole plant responses to salinity are also emerging as an important Research Topic. Feng et al. reviewed that soybean is an important economic crop whose productivity is hampered by abiotic stress, notably salt stress. They concluded that salt stress sensing, signaling, ionic balance (Na^+^/K^+^), and osmotic stress compensation may all play a role in controlling the soybean salt stress response. Applying traditional transgenic and cutting-edge breeding techniques to create salt-tolerant soybean plants can help reduce salinity stress. Huge genetic diversity and excellent soybean genome accessibility aid in the identification of salinity stress-responsive genetic signals, molecular networks, and QTLs. Plant virus-induced autophagy has recently been extensively examined (Yang and Liu, 2022). Furthermore, hydroxysteroid dehydrogenases (HSDs) are crucial in regulating various abiotic stresses, including salt stress (Sameeullah et al., 2021). Saleem et al. conducted a GWI and analysis of a phylogenetic tree comparing different HSDs in ranging from lower to higher plants. HSD1 was found in nearly all plants except for algae, while HSD2 was found in both monocot and dicot plants, and HSD5 was only found in land plants. Their research shows that the HSD1s and HSD5s in dicot plants are mostly acidic, and the HSD1s and HSD2s in monocot plants are mostly basic. Cis-regulatory elements and expression analysis suggested that *HSDs* in plants may play roles in a variety of abiotic stresses.

In addition to abiotic stress, plants also experience a wide range of other biotic stresses. Qing et al. found that PVY infection led to the downregulation of ATG6, which thus hindered the breakdown of NIb proteins. The findings indicate that the interaction between P3/P3N-PIPO of PVY and BI-1 enhances virus proliferation by preventing the NIb damage. Plants undergoing acidification stress could modify the absorption of soil elements. The processes by which plants utilize and absorb these elements vary significantly across different types of abiotic stress (Dhaliwal et al., 2022).

Soil acidification is a limitation that involved in declining tea productivity and quality (Jia et al.). In this study, a total of 59 hormones identified in tea leaves were altered their levels due to varying soil pH levels. Also, they found that elevating soil pH (3.29–5.32) improved the nutrient content of rhizosphere soil, antioxidant enzyme activity, photosynthetic capacity, and growth. An increase in soil pH led to the accumulation of key elements in tea tree leaves and promoted the synthesis of key hormones as well. In addition, several molecular alterations occur under stress response in plants. Ubiquitination is a key regulator process of protein homeostasis, wherein it serving to remove injured or unwanted proteins in response to environmental stress. It represents a form of protein breakdown and post-translational modification, which can be reversed by deubiquitinase (DUBs). This process plays a significant role in various biological processes such as plant growth, cell cycle regulation, and response to environmental stressors. Tang et al. investigated DUBs in potato diploid DM and tetraploid Atlantic and C88, utilizing Atlantic as the reference genome for initial transcriptome sequencing analysis. Their research highlights the response of potato deubiquitinating enzymes to osmotic stress, providing valuable genetic insights for studying tetraploid potatoes.

The latest publication in this research area aids readers in developing a deeper understanding of how hormones and biostimulants are linked to stress responses and their impact on plant growth and productivity. However, the latest findings of this Research Topic opens plant hormone and biostimulant based environmental friendly strategies with new perspectives in developing plant growth, fitness and stress tolerance for sustainable agricultural production with global food security.

## Author contributions

MS: Conceptualization, Writing – original draft. MH: Conceptualization, Writing – review & editing. MR: Conceptualization, Writing – review & editing.
